# Role of Single Low Pulse Intensity of Transcranial Magnetic Stimulation Over the Frontal Cortex for Cognitive Function

**DOI:** 10.3389/fnhum.2020.00205

**Published:** 2020-07-03

**Authors:** Shahid Bashir, Fawaz Al-Hussain, Ali Hamza, Ghadah Faisal Shareefi, Turki Abualait, Woo-Kyoung Yoo

**Affiliations:** ^1^Department of Neurophysiology, Neuroscience Center, King Fahad Specialist Hospital, Dammam, Saudi Arabia; ^2^Department of Neurology, College of Medicine, King Saud University, Riyadh, Saudi Arabia; ^3^Department of Electrical Engineering, National University of Computer and Emerging Sciences, Lahore, Pakistan; ^4^College of Applied Medical Sciences, Imam Abdulrahman Bin Faisal University, Dammam, Saudi Arabia; ^5^Department of Physical Medicine and Rehabilitation, Division of Neuroscience Center, Hallym University Sacred Heart Hospital, Anyang, South Korea

**Keywords:** monophasic TMS, neuromodulation, subthreshold TMS, dorsolateral prefrontal cortex, cognitive functions

## Abstract

**Background**: The principal aim of this study was to measure the effect of online single-pulse transcranial magnetic stimulation (TMS) over the right dorsolateral prefrontal cortex (DLPFC) on cognition *via* the Cambridge Neuropsychological Test Automated Battery (CANTAB) in healthy individuals.

**Methods**: In a single-blind, sham-controlled study, we assessed both 50% and 60% of the resting motor threshold (RMT) over the right DLPFC in healthy right-handed (*n* = 42) adults using cognitive function, such as attention and memory, as a measure *via* CANTAB.

**Results**: We observed an improvement in the cognitive function level during the use of online low intensities of 50% and 60% RMT active stimulation of the DLPFC compared to the sham stimulation.

**Conclusions**: The results showed that low-intensity TMS can indeed effectively modulate cognitive function in DLPFC. Future research is, however, necessary to investigate the potential effects of low-intensity TMS on different brain areas to increase confidence in the observed results.

## Background

Transcranial magnetic stimulation (TMS) is a noninvasive brain stimulation method that has become increasingly used in basic neuroscience research (Alford et al., 2007; Balslev et al., 2007) and has been evaluated as a possible therapeutic intervention in some neurological and psychiatric disorders (Cotelli et al., 2008; Bashir et al., 2010).

The era of using TMS to study cognitive functions began with the classic work of Amassian et al. (1989), who performed experiments over the visual cortex in human subjects to impair the participants’ ability to report briefly presented letters (Bliss et al., 2003).

The literature includes reports of TMS either improving or impairing cognitive performance in terms of response time (RT; Boroojerdi et al., 2001; Drager et al., 2004) and memory tasks (Cappa et al., 2002; Luber et al., 2007a; Boyd and Linsdell, 2009). Previous work on TMS has presented conflicting results in terms of increasing or decreasing cognitive performance in task-related perception, attention, conceptualization, memory, reasoning, and motor performance (Pascual-Leone and Hallett, 1994; Evers et al., 2001; Cotelli et al., 2006, 2011; Cattaneo et al., 2008, 2009a,b, 2010). In this study, we focused mainly on the effects of low-intensity online TMS *via* a single pulse over the frontal cortex.

The prefrontal cortex, specifically the dorsolateral prefrontal cortex (DLPFC), of the brain is one of the most interesting repetitive transcranial magnetic stimulation (rTMS) regions for implementing therapeutic interventions for neuropsychiatric disorders, including major depression (Feredoes et al., 2007; Gagnon et al., 2011). Cognitive task-related working memory (WM) and executive function have shown functioning in the DLPFC region (Hamidi et al., 2009b; Galea et al., 2010). Interestingly, a single session of rTMS in this region showed improvement in cognitive task-related response inhibition (Hamidi et al., 2009a), mental rotation (Hamidi et al., 2011), and confrontation naming (Hannula et al., 2010). Neuroimaging methods can highlight the roles of neuronal priming, oscillatory activity, and synaptic neuroplastic changes in relation to the cognitive task (Koch et al., 2005; Köhler et al., 2006; Luber et al., 2007b; Kobayashi et al., 2009).

WM tasks have been studied extensively using single-pulse TMS and rTMS (Cotelli et al., 2006, 2011; Cattaneo et al., 2008, 2009a,b) to investigate the cognitive process when a limited amount of information is provided for a brief period. In some TMS studies, both accuracy and slowing of the RT were used to measure the impairment of performance (Pascual-Leone and Hallett, 1994; Evers et al., 2001; Cotelli et al., 2006, 2011; Feredoes et al., 2007; Cattaneo et al., 2008, 2009a,b, 2010; Galea et al., 2010; Gagnon et al., 2011).

We conducted the current study to examine the effects of online single-pulse, low-intensity TMS of the frontal cortex in healthy individuals regarding their neuropsychological abilities *via* using the Cambridge Neuropsychological Test Automated Battery (CANTAB) to measure their performance on cognitive tasks.

It is reasonable to suggest that a single TMS pulse will induce a distinct temporal sequence of facilitation and suppression at a given cell (Romero et al., 2019), which will be related either to direct stimulation of the cell or, more likely, in other cortical structures, to the activation of excitatory or inhibitory inputs within the local network. This study investigated the neural effects of low-intensity TMS on performance (Romero et al., 2019). We used monophasic, single-pulse TMS with intensities far below previously reported intensities (50% and 60% MT). Due to the low intensity of the TMS used, we hypothesized modulating effects over the stimulated area and thus changes in performance on the Stop Signal Task (SST) and Pattern Recognition Memory (PRM), which were assessed *via* CANTAB.

## Materials and Methods

### Participants

We recruited 42 participants, including 24 males and 18 females with mean ages of 25.6 and 26.1, respectively, to participate in a single-blind online sham or active-stimulation measured study. The random sequence generator (https:random.org 2017) was used to randomly allocate each subject to one of the three conditions (active 50%, active 60%, and the sham stimulation).

All participants had either normal or corrected-to-normal visual acuities, and they were screened for risk factors of the noninvasive brain stimulation application through safety questionnaires. All participants were naive to TMS stimulation, and none were taking medication or had a history of neurological or psychiatric disorders. Written informed consent was obtained from each participant before the experiment, which was approved by the IRB of King Saud University.

### Procedure and Materials

The demographic information and the safety of the TMS questionnaires were completed in the screening section. The participants then performed cognitive function tasks using the CANTAB research suite software (version 6.0.37, Cambridge Cognition, Cambridge, UK) during single-pulse TMS stimulation.

#### Stop Signal Test

The SST measured response inhibition (impulse control) *via* each participant’s response to an arrow stimulus of two choices, depending on the direction in which the arrow was pointing on the touchscreen, and mental processing speed. The subject had to inhibit their response when an audio tone sounded during the task. Therefore, this test comprised two parts.

(1) When each subject was presented with a stimulus of a left-pointing arrow on the screen, they were told to press the left button. When they were presented with a right-pointing arrow, they were told to press the right button. There was one block of 16 trials for the participant to practice this task.
(2) The participant was told to continue pressing the buttons on the press pad when they saw the arrows, as before; however, they were told that, if they heard an auditory signal (a beep), they should withhold their response and not press the button.

#### Pattern Recognition Memory

PRM is a two-choice, forced-discrimination paradigm that is used to test visual PRM. In the task, a sequence of visual patterns, which could not be easily described *via* verbal labels, was presented in the center of a screen. In the recognition phase, the subjects were required to choose between a pattern that they had already seen and a novel pattern.

### Experimental Procedure

During the experiment, the subjects were seated in a height-adjustable chair, and their head position was fixed by a chinrest at a distance of 1 m in front of a computer screen. The stimulation setup consisted of a frameless stereotaxic system for navigation (Visor2 ANT; Netherlands). A brain MRI of each subject was used to ensure stimulation accuracy both during and across the sessions. Co-registration errors to the MRI’s surface landmarks were matched to ≤3 mm at each follow-up session. During the delivery of TMS, the 3D brain image of the participant’s cortex and the hot spot were visualized with brain stimulation software for optimal delivery. Individually determined positions (DLPFC) were used during all TMS conditions for guidance of the coil to ensure consistency of the TMS application throughout the experiment.

Prior to the experiment, the individual resting motor threshold (RMT) was obtained at the right abductor pollicis brevis muscle by delivering TMS to the contralateral (left) M1. The RMT was determined as the minimum intensity needed for eliciting motor-evoked potentials of at least 50 μV in amplitude (baseline to peak) in at least four of eight single TMS pulses. The participants were asked to relax their hand muscles and to keep their eyes open during the procedure.

During the SST and PRM, TMS was applied at the visual stimulus onset over the right DLPFC. The intensity of the TMS pulses was set to 50% and 60% of the individual RMT, respectively. Additionally, the sham TMS was applied by tilting the coil 90% away from the head so that only the outer edge of the coil was attached to the subject’s head. The stimulator output during the sham was set to 50% of the individual RMT. The monophasic single-pulse TMS delivered 320 stimuli, with an overall duration of 1 ms between stimuli.

#### Analysis

The SST had five outcome measures: covered direction errors, the proportion of successful stops, the RT on the GO trials, SSD (50%), and the stop signal reaction time (SSRT). A statistical comparison was performed using an ANOVA with the factors’ stimulation intensity (50%, 60%, and sham). Regarding the significant main effects, *post hoc* paired-sample *t*-tests were calculated between intensities. A significance threshold of *p* < 0.05 was applied. The normal distribution of the data was tested using the one-sample Kolmogorov–Smirnov test over all conditions and learning stages.

## Results

All data were normally distributed and thus fulfilled the requirements for parametrical testing. The mean intensity (±SD) of the RMT over all sessions was 42.5% (±6.4%), with a range between 34% and 46% of the maximal stimulator output (Table 1). A repeated-measures ANOVA found no significant difference in the individual RMTs between the sessions (*F* = 2.20, *p* > 0.05).

**Table 1:** Distribution of participants across conditions and experiments.

	Experiment 1	Experiment 2	Experiment 3
TMS intensity	50% RMT	60% RMT	Sham
Participants (*n*)	14	14	14
Resting motor threshold (RMT)	43 ± 7.2	42 ± 8.1	42 ± 5.7
Sex (M/F)	8/6	9/5	7/7
Age (years mean ± SD)	26.5 ± 4.64	25.7 ± 4.86	26.6 ± 3.88

*RMT, resting motor threshold*.

**Figure 1:**
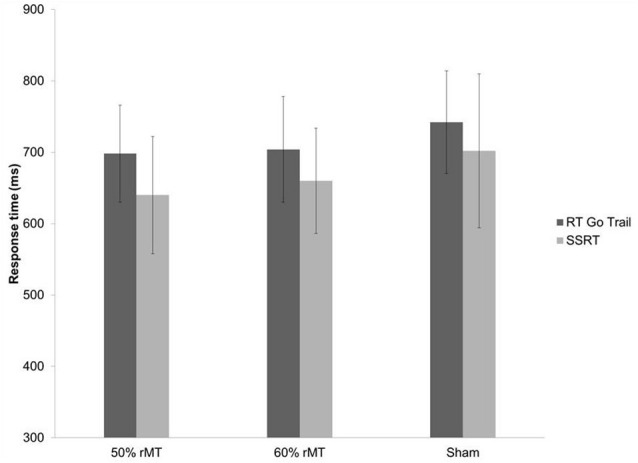
Comparison of Stop Signal Task (SST) for mean reaction time (RT) on GO trial, and stop signal reaction time (SSRT) during 50% and 60% of resting motor threshold (RMT) and sham stimulation for the right dorsolateral prefrontal cortex (DLPFC). Error bars are standard deviation.

Low-intensity TMS induced changes in performance of the RT on the GO trials and the SSRT. The ANOVA showed a significant main effect of the TMS’ intensity on the RT for the GO trials [*F* = 4.61, *p* < 0.05 (0.037)] and for the SSRT [*F* = 4.06, *p* < 0.05 (0.041)]. There was also a trend toward significance of the intensity on the RT for the GO trials [*F* = 3.71, *p* < 0.1 (0.052)] and for the SSRT [*F* = 3.86, *p* < 0.05 (0.05)]. There was no significant difference in direction errors and the proportion of successful stops in the SST. The *post hoc* paired-sample *t*-tests between the intensity revealed significantly faster reaction times at 50% [mean ± SD (ms)] for the RT during the GO trials (698.12 ± 68.20) and the SSRT (640.90 ± 82.16) compared to those of the sham [mean ± SD (ms)] at 742.20 ± 72.1 for the RT during the GO trials and 702.04 ± 108.18 for the SSRT, with *t* = −4.12, *p* < 0.05 and *t* = −3.88, *p* < 0.05, respectively. The results showed faster reaction times at 60% [mean ± SD (ms)] for the RT during the GO trials and the SSRT (704.14 ± 74.4 and 660.42 ± 84.57, respectively), compared to those of the sham, with *t* = −2.92, *t* = −3.02, *p* < 0.05, respectively (Figure 1).

There was no significant difference between the 50% and 60% RMT intensity (*p* < 0.05) for any SST measurements.

**Figure 2:**
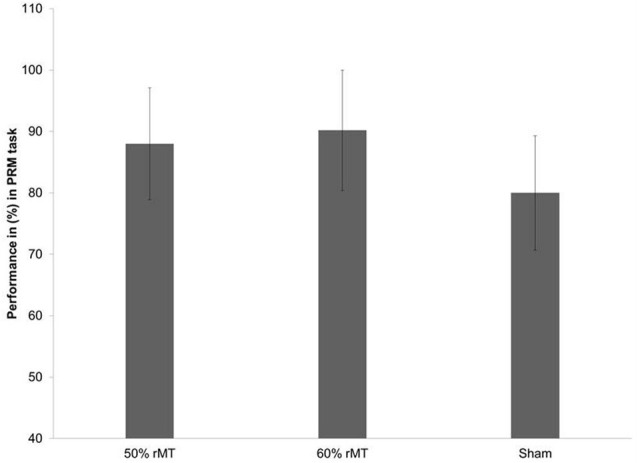
Comparison of pattern recognition task performance in % during 50% and 60% of RMT and sham stimulation for the right DLPFC. Error bars are standard deviation.

The ANOVA showed a significant main effect for TMS intensity in the PRM correct trials [*F* = 4.02, *p* < 0.05 (0.04)]. The *post hoc* paired-sample *t*-tests between the intensity revealed significantly improved PRM performance at 50% [mean ± SD (%): 88.8 ± 9.1] as well as better performance at 60% [mean ± SD (%): 90.2 ± 9.8; *t* = −3.76, *p* < 0.05] compared to the sham [mean ± SD (%): 80.7 ± 9.3; *t* = −3.06, *p* < 0.05; Figure 2].

## Discussion

This study investigated the neuropsychological alterations of a cohort of healthy individuals with online single-pulse, low intensities of 50% and 60% of the RMT TMS protocol on the DLPFC compared to a sham group. The findings showed that low-intensity TMS significantly impacted the cognitive performance of the RT during the GO trials and the SSRT. There was no significant improvement in PRM compared to the sham group. Considering these results, it can be suggested that subthreshold single-pulse TMS might maintain its conditioning abilities if, instead of a second TMS pulse, upcoming neural activation would reach the modulated area during “normal” neural processing. If this suggestion is correct, a conditioning subthreshold TMS pulse could lead to a change in excitability in a target area, which might result in changes in performance. Previous work with lower intensities of TMS showed a similar pattern of enhanced performance from the baseline level (Pascual-Leone and Hallett, 1994; Balslev et al., 2007; Cotelli et al., 2008; Silvanto and Muggleton, 2008; Silvanto et al., 2008b; Oliveri et al., 2010). In particular, the role of the DLPFC was studied while participants were performing an SST. The DLPFC was activated when the two nonassociated concepts required the same response, indicating a need for the cognitive control of inhibition and excitation (Pascual-Leone et al., 1994; Thut et al., 2005; Luber et al., 2007a; Cattaneo et al., 2010). The mechanism of inhibition and excitation in the cortical structure for a single TMS pulse is not clear; it is likely that, in cortical structures, there is activation of excitatory or inhibitory inputs within the local network for task performance (Luber et al., 2007a; Cattaneo et al., 2010; Pascual-Leone et al., 1994). The strength of the TMS stimulus might be affected by the functional state of the network to produce performance enhancements (Cattaneo et al., 2009a,b; Hannula et al., 2010; Thut et al., 2011). Low-intensity, single-pulse TMS for a targeted region should arguably represent a similar pattern in individuals with lesions or damage in that target area. This study provides further evidence that the DLPFC is involved in some aspect of cognitive stereotyping of the mental response. These results have important implications for the involvement of the DLPFC in the neural networks that are involved in response inhibition stereotypes. The findings suggest that the additional neural activity that is caused by the TMS pulse may often have brought the neural response to the target stimulus above the threshold of awareness. Nonetheless, these findings should be interpreted in consideration of the high stimulation protocol variability of the investigated studies (Van Der Werf and Paus, 2006). Although our results are unique in the sense that the test included either 50% or 60% subthreshold conditioning stimuli prior to the task performance, it is difficult to exclude the variability of the stimulation coil and the stimulation site as well as the responding time of the individual task session in this outcome because no physiological measure, such as an EEG, was obtained. In a previous study, the use of a monophasic (unidirectional) single-pulse TMS-activated one neuronal population was oriented in the same direction, which summated their effects and thus yielded a stronger short-term effect (Knutson et al., 2007).

The findings of the current study might be attributed to the TMS effects on remote areas, which can be observed after a period of stimulation. Vink et al. (2018) applied single pulses of TMS with an intensity of 60% RMT over the DLPFC in healthy subjects using a TMS-fMRI setup. They found that when they delivered TMS pulses with such intensity, it induced neuronal activity (increased the BOLD fMRI signal) in several connected brain regions, including the subungual anterior cingulate cortex (ACC). The ACC might be of particular interest because this region, along with other prefrontal cortex areas, has been found to be engaged by attentional and memory processes (Kim, 2014; Duverne and Koechlin, 2017). Interestingly, it has been reported that the ACC has a significant role in inhibiting the ongoing task set, which can be observed in the SST (Boorman et al., 2013). This may indicate that the effects of stimulation over the DLPFC might be propagated to other brain regions, providing a potential neural substrate for processing the PRM task and response inhibition during the SST (Kim, 2014).

Similarly, in a recent study, Romero et al. (2019) showed that the effect of single-pulse TMS (with a low-intensity 60% RMT) had a limited spatial extent of TMS-induced spiking activity of individual neurons in awake behaving monkeys. Notably, it was observed that the spread of the single-pulse TMS-evoked spikes was more focal, while the TMS-induced oscillatory activity spread more remotely.

Taken together, these findings might highlight that the effect of the single-pulse TMS in the current study is due to oscillatory neuronal activity that influenced other brain areas remotely, such as the ACC, which resulted in enhancing cognitive performance.

## Limitation of Study

A limitation of this study was that only monophasic, single-pulse TMS with two low intensities (50% and 60% RMT) was employed. Moreover, the investigated cognitive functions in this study were limited to only the attention and memory domains. Using different types or intensities of single-pulse TMS would provide different findings. Further studies on a larger population size to confirm the efficacy of low-intensity TMS on different brain regions, including the parietal and cerebral regions, should be done. In addition, our study’s age range was too broad, considering that cognitive function could be different according to age. Finally, single-pulse TMS was used to study functional connectivity in the brain because stimulation in one area has been shown to affect blood flow in connected areas (Paus et al., 1997). Thus, it is likely that the TMS protocol used here not only impacted the DLPFC but also affected functionally connected areas as well. Nonetheless, this does not undermine our central conclusions that the DLPFC is part of a functional network representing information that measured both response inhibition and mental processing speed.

## Conclusion

The current work shows that online low-intensity TMS can influence executive function and cognitive and visuospatial associative learning behavior. This creates an exciting opportunity for developing this approach as a therapeutic intervention, especially for a group of DLPFC disorders that lack effective alternative treatments. However, we are still in the process of understanding much of the properties of low-intensity TMS and how best to apply this technique to human subjects.

## Data Availability Statement

All data are included in the article. However, the datasets used and/or analyzed during the current study are available from the corresponding author on reasonable request.

## Ethics Statement

The protocol of research was reviewed and approved by the Ethics and Research Committee of the King Saud University, and written consent was obtained from all participants before starting the procedure.

## Author Contributions

All listed authors developed different substantial activities. SB performed the main techniques of the experiment and interpretation of data. FA-H and AH analyzed the data, and they were involved in drafting the manuscript. GF and FA-H contributed substantially to the experiment design. TA participated in drafting and writing the manuscript. SB, AH, and W-KY did the statistical analysis of the results, and they participated in drafting and writing the manuscript. Each author participated sufficiently in writing and reviewing the manuscript. All authors read and approved the final manuscript.

## Conflict of Interest

The authors declare that the research was conducted in the absence of any commercial or financial relationships that could be construed as a potential conflict of interest.

## Abbreviations

DLPFC: right dorsolateral prefrontal cortex
CANTAB: Cambridge Neuropsychological Test Automated Battery
RMT: Resting motor threshold
TMS: transcranial magnetic stimulation
rTMS: repetitive transcranial magnetic stimulation
RT: response time
SST: Stop Signal Task
PRM: Pattern Recognition Memory
SSRT: stop signal reaction time.

## References

[B1] Alford J. L. Van Donkelaar P. Dassonville P. Marrcco R. T. (2007). Transcranial magnetic stimulation over MT/MST fails to impair judgments of implied motion. Cogn. Affect. Behav. Neurosci. 7, 225–232. 10.3758/cabn.7.3.225 17993208

[B4100] Amassian V. E. Cracco R. Q. Maccabee P. J. Cracco J. B. Rudell A. Eberle L. (1989). Suppression of visual perception by magnetic coil stimulation of human occipital cortex. Electroenceph. Clin. Neurophysiol./Evoked Potentials Section 74, 458–462. 10.1016/0168-5597(89)90 2480226

[B2] Balslev D. Braet W. McCallister C. Miall R. C. (2007). Inter-individual variability in optimal current direction for transcranial magnetic stimulation of the motor cortex. J. Neurosci. Methods 162, 309–313. 10.1016/j.jneumeth.2007.01.021 17353054

[B3] Bashir S. Mizrahi I. Weaver K. Fregni F. Pascual-Leone A. (2010). Assessment and modulation of neural plasticity in rehabilitation with transcranial magnetic stimulation. PMR 2, S253–S268. 10.1016/j.pmrj.2010.10.015 21172687 PMC3951769

[B4] Bliss T. V. Collingridge G. L. Morris R. G. (2003). Introduction. Longterm potentiation and structure of the issue. Philos. Trans. R. Soc. Lond. B Biol. Sci. 358, 607–611. 10.1098/rstb.2003.1282 12740102 PMC1693168

[B5] Boorman E. D. Rushworth M. F. Behrens T. E. (2013). Ventromedial prefrontal and anterior cingulate cortex adopt choice and default reference frames during sequential multi-alternative choice. J. Neurosci. 33, 2242–2253. 10.1523/jneurosci.3022-12.2013 23392656 PMC3743024

[B6] Boroojerdi B. Phipps M. Kopylev L. Wharton C. M. Cohen L. G. Grafman J. (2001). Enhancing analogic reasoning with rTMS over the left prefrontal cortex. Neurology 56, 526–528. 10.1212/wnl.56.4.526 11222799

[B7] Boyd L. A. Linsdell M. A. (2009). Excitatory repetitive transcranial magnetic stimulation to left dorsal premotor cortex enhances motor consolidation of new skills. BMC Neurosci. 10:72. 10.1186/1471-2202-10-72 19583831 PMC2713248

[B8] Cappa S. F. Sandrini M. Rossini P. M. Sosta K. Miniussi C. (2002). The role of the left frontal lobe in action naming: rTMS evidence. Neurology 59, 720–723. 10.1212/wnl.59.5.720 12221163

[B10] Cattaneo Z. Rota F. Vecchi T. Silvanto J. (2008). Using state-dependency of transcranial magnetic stimulation (TMS) to investigate letter selectivity in the left posterior parietal cortex: a comparison of TMS-priming and TMS-adaptation paradigms. Eur J. Neurosci. 28, 1924–1929. 10.1111/j.1460-9568.2008.06466.x 18973605

[B11] Cattaneo Z. Rota F. Walsh V. Vecchi T. Silvanto J. (2009a). TMS-adaptation reveals abstract letter selectivity in the left posterior parietal cortex. Cereb. Cortex 19, 2321–2325. 10.1093/cercor/bhn249 19150919

[B12] Cattaneo Z. Vecchi T. Pascual-Leone A. Silvanto J. (2009b). Contrasting early visual cortical activation states causally involved in visual imagery and short-term memory. Eur. J. Neurosci. 30, 1393–1400. 10.1111/j.1460-9568.2009.06911.x 19788574

[B9] Cattaneo L. Sandrini M. Schwarzbach J. (2010). State-dependent TMS reveals a hierarchical representation of observed acts in the temporal, parietal and premotor cortices. Cereb. Cortex 20, 2252–2258. 10.1093/cercor/bhp291 20051360

[B13] Cotelli M. Calabria M. Manenti R. Rosini S. Zanetti O. Cappa S. F.. (2011). Improved language performance in Alzheimer disease following brain stimulation. J. Neurol. Neurosurg. Psychiatry 82, 794–797. 10.1136/jnnp.2009.197848 20574108

[B14] Cotelli M. Manenti R. Cappa S. F. Geroldi C. Zanetti O. Rossini P. M.. (2006). Effect of transcranial magnetic stimulation on action naming in patients with Alzheimer disease. Arch. Neurol. 63, 1602–1604. 10.1001/archneur.63.11.1602 17101829

[B15] Cotelli M. Manenti R. Cappa S. F. Zanetti O. Miniussi C. (2008). Transcranial magnetic stimulation improves naming in Alzheimer disease patients at different stages of cognitive decline. Eur. J. Neurol. 15, 1286–1292. 10.1111/j.1468-1331.2008.02202.x 19049544

[B16] Drager B. Breitenstein C. Helmke U. Kamping S. Knecht S. (2004). Specific and nonspecific effects of transcranial magnetic stimulation on picture-word verification. Eur. J. Neurosci. 20, 1681–1687. 10.1111/j.1460-9568.2004.03623.x 15355336

[B17] Duverne S. Koechlin E. (2017). Rewards and cognitive control in the human prefrontal cortex. Cereb. Cortex 27, 5024–5039. 10.1093/cercor/bhx210 28922835

[B18] Evers S. Bockermann I. Nyhuis P. W. (2001). The impact of transcranial magnetic stimulation on cognitive processing: an event-related potential study. Neuroreport 12, 2915–2918. 10.1097/00001756-200109170-00032 11588602

[B19] Feredoes E. Tononi G. Postle B. R. (2007). The neural bases of the short-term storage of verbal information are anatomically variable across individuals. J. Neurosci. 27, 11003–11008. 10.1523/JNEUROSCI.1573-07.2007 17928441 PMC6672855

[B20] Gagnon G. Schneider C. Grondin S. Blanchet S. (2011). Enhancement of episodic memory in young and healthy adults: a paired-pulse TMS study on encoding and retrieval performance. Neurosci. Lett. 488, 138–142. 10.1016/j.neulet.2010.11.016 21094215

[B21] Galea J. M. Albert N. B. Ditye T. Miall R. C. (2010). Disruption of the dorsolateral prefrontal cortex facilitates the consolidation of procedural skills. J. Cogn. Neurosci. 22, 1158–1164. 10.1162/jocn.2009.21259 19413472 PMC6010144

[B22] Hamidi M. Johnson J. S. Feredoes E. Postle B. R. (2011). Does high-frequency repetitive transcranial magnetic stimulation produce residual and/or cumulative effects within an experimental session? Brain Topogr. 23, 355–367. 10.1007/s10548-010-0153-y 20623171 PMC2978750

[B23] Hamidi M. Slagter H. A. Tononi G. Postle B. R. (2009a). Repetitive transcranial magnetic stimulation affects behavior by biasing endogenous cortical oscillations. Front. Integr. Neurosci. 3:14. 10.3389/neuro.07.014.2009 19587850 PMC2707056

[B24] Hamidi M. Tononi G. Postle B. R. (2009b). Evaluating the role of prefrontal and parietal cortices in memory-guided response with repetitive transcranial magnetic stimulation. Neuropsychologia 47, 295–302. 10.1016/j.neuropsychologia.2008.08.026 18822306 PMC2704005

[B25] Hannula H. Neuvonen T. Savolainen P. Hiltunen J. Ma Y.-Y. Antila H.. (2010). Increasing top-down suppression from prefrontal cortex facilitates tactile working memory. NeuroImage 49, 1091–1098. 10.1016/j.neuroimage.2009.07.049 19643184

[B26] Kim H. (2014). Involvement of the dorsal and ventral attention networks in oddball stimulus processing: a meta-analysis. Hum. Brain Mapp. 35, 2265–2284. 10.1002/hbm.22326 23900833 PMC6868981

[B27] Knutson K. M. Mah L. Manly C. F. Grafman J. (2007). Neural correlates of automatic beliefs about gender and race. Hum. Brain Mapp. 28, 915–930. 10.1002/hbm.20320 17133388 PMC6871386

[B28] Kobayashi M. Theoret H. Pascual-Leone A. (2009). Suppression of ipsilateral motor cortex facilitates motor skill learning. Eur. J. Neurosci. 29, 833–836. 10.1111/j.1460-9568.2009.06628.x 19200062 PMC2771229

[B29] Koch G. Oliveri M. Torriero S. Carlesimo G. A. Turriziani P. Caltagirone C. (2005). rTMS evidence of different delay and decision processes in a fronto-parietal neuronal network activated during spatial working memory. NeuroImage 24, 34–39. 10.1016/j.neuroimage.2004.09.042 15588594

[B30] Köhler S. Paus T. Buckner R. L. Milner B. (2006). Effect of left inferior prefrontal stimulation on episodic memory formation: a two-stage fMRI-rTMS study. J. Cogn. Neurosci. 16, 178–188. 10.1162/089892904322984490 15068590

[B31] Luber B. Balsam P. Nguyen T. Gross M. Lisanby S. H. (2007a). Classical conditioned learning using transcranial magnetic stimulation. Exp. Brain Res. 183, 361–369. 10.1007/s00221-007-1052-7 17639360

[B32] Luber B. Kinnunen L. H. Rakitin B. C. Ellsasser R. Stern Y. Lisanby S. H. (2007b). Facilitation of performance in a working memory task with rTMS stimulation of the precuneus: frequency and time-dependent effects. Brain Res. 1128, 120–129. 10.1016/j.brainres.2006.10.011 17113573

[B33] Oliveri M. Zhaoping L. Mangano G. R. Turriziani P. Smirni D. Cipolotti L. (2010). Facilitation of bottom-up feature detection following rTMS-interference of the right parietal cortex. Neuropsychologia 48, 1003–1010. 10.1016/j.neuropsychologia.2009.11.024 20025892

[B34] Pascual-Leone A. Hallett M. (1994). Induction of errors in a delayed response task by repetitive transcranial magnetic stimulation of the dorsolateral prefrontal cortex. Neuroreport 5, 2517–2520. 10.1097/00001756-199412000-00028 7696593

[B35] Pascual-Leone A. Valls-Sole J. Wassermann E. M. Hallett M. (1994). Responses to rapid-rate transcranial magnetic stimulation of the human motor cortex. Brain 117, 847–858. 10.1093/brain/117.4.847 7922470

[B36] Paus T. Jech R. Thompson C. J. Comeau R. Peters T. Evans A. C. (1997). Transcranial magnetic stimulation during positron emission tomography: a new method for studying connectivity of the human cerebral cortex. J. Neurosci. 17, 3178–3184. 10.1523/JNEUROSCI.17-09-03178.1997 9096152 PMC6573635

[B37] Romero M. C. Davare M. Armendariz M. Janssen P. (2019). Neural effects of transcranial magnetic stimulation at the single-cell level. Nat. Commun. 10:2642. 10.1038/s41467-019-10638-7 31201331 PMC6572776

[B38] Silvanto J. Cattaneo Z. Batteli L. Pascual-Leone A. (2008b). Baseline cortical excitability determines whether TMS disrupts or facilitates behavior. J. Neurophysiol. 99, 2725–2730. 10.1152/jn.01392.2007 18337360 PMC3533239

[B39] Silvanto J. Muggleton N. (2008). New light through old windows: moving beyond the “virtual lesion” approach to transcranial magnetic stimulation. NeuroImage 39, 549–552. 10.1016/j.neuroimage.2007.09.008 17945512

[B40] Thut G. Nietzel A. Pascual-Leone A. (2005). Dorsal posterior parietal rTMS affects voluntary orienting of visuospatial attention. Cereb. Cortex 15, 628–638. 10.1093/cercor/bhh164 15342434

[B41] Thut G. Veniero D. Romei V. Miniussi C. Schyna P. Gross J. (2011). Rhythmic TMS causes local entrainment of natural oscillatory signatures. Curr. Biol. 21, 1176–1185. 10.1016/j.cub.2011.05.049 21723129 PMC3176892

[B42] Van Der Werf Y. D. Paus T. (2006). The neural response to transcranial magnetic stimulation of the human motor cortex. I. Intracortical and cortico-cortical contributions. Exp. Brain Res. 175, 231–245. 10.1007/s00221-006-0551-2 16783559

[B43] Vink J. J. T. Mandija S. Petrov P. I. van den Berg C. A. T. Sommer I. E. C. Neggers S. F. W. (2018). A novel concurrent TMS-fMRI method to reveal propagation patterns of prefrontal magnetic brain stimulation. Hum. Brain Mapp. 39, 4580–4592. 10.1002/hbm.24307 30156743 PMC6221049

